# Parkin regulates NF-κB by mediating site-specific ubiquitination of RIPK1

**DOI:** 10.1038/s41419-018-0770-z

**Published:** 2018-06-28

**Authors:** Yu Wang, Bing Shan, Yaosi Liang, Huiting Wei, Junying Yuan

**Affiliations:** 1 Department of Cell Biology, Harvard Medical School, 240 Longwood Ave., Boston, MA 02115 USA; 2 South China Sea Resource Exploitation and Protection Collaborative Innovation Center, Sun Yat-sen University, Guangzhou, 510275 P. R. China; 3 State Key Laboratory of Biocontrol, Institute of Aquatic Economic Animals and Guangdong Provincial Key Laboratory for Aquatic Economic Animals, College of Life Sciences, Sun Yat-sen University, Guangzhou, 510275 China; 4 Interdisciplinary Research Center on Biology and Chemistry, Shanghai Institute of Organic Chemistry, Chinese Academy of Sciences, Shanghai, China

## Abstract

Parkin (Park2), a RING-between-RING-type E3 ubiquitin ligase, has been implicated in regulating NF-κB. Mutations in Parkin are associated with Parkinson’s disease. Here we investigated the interaction of Parkin with Receptor-interacting protein kinase 1 (RIPK1) kinase, a key mediator of multiple signaling pathways activated by TNFR1 including NF-κB pathway. We report that Parkin interacts with RIPK1 and mediates K63 ubiquitination of RIPK1 on K376 in TNFR1-signaling pathway. The expression of Parkin promotes the recruitment of transforming growth factor β (TGF-β)-activated kinase 1 (TAK1), nuclear factor-κB (NF-κB) essential molecule (NEMO), Sharpin and A20 in complex I associated with TNFR1 upon TNFα stimulation. Ubiquitination of RIPK1 by Parkin increases the activation of NF-κB and mitogen-activated protein kinases (MAPKs) by promoting the phosphorylation of inhibitor of kappa B kinase (IKK)α/β and IκBα and nuclear translocation of p65. Thus, we conclude that Parkin modulates the K63 ubiquitination status of RIPK1 to promote the activation of NF-κB and MAPKs.

## Introduction

Receptor-interacting protein kinase 1 (RIPK1) is a key mediator of multiple signaling pathways downstream of tumor necrosis factor (TNF) receptor 1 (TNFR1) in promoting inflammation and cell death^1, 2^. Activation of TNFR1 by TNFα leads to the rapid recruitment of RIPK1, TNF receptor-associated death domain (TRADD), TNF receptor-associated factor 2 (TRAF2) and E3 ubiquitin ligases, cellular inhibitor of apoptosis protein 1/2 (cIAP1/2), to form complex I, also called TNFR1-signaling-complex (TNF-RSC), associated with the intracellular domain of TNFR1. The scaffold function of RIPK1 is involved in mediating the activation of nuclear factor-κB (NF-κB) after its ubiquitination at K376 residue by cIAP1/2^3–5^. K63 polyubiquitin chains on RIPK1 and other components of complex I function as anchors in the recruitment of the key activators of NF-κB pathway, such as the transforming growth factor β (TGF-β)-activated kinase 1 (TAK1) binding protein 1/2 (TAB1/2) and NEMO, to promote the activation of TAK1 and IKK complex, respectively^3, 6, 7^. Activation of TAK1 is important not only for activating NF-κB pathway but also for suppressing RIPK1 kinase by phosphorylation of multiple residues on RIPK1 such as S321^8^. K63 ubiquitination of complex I is important for the recruitment of the linear ubiquitin assembly complex (LUBAC), consisting of heme-oxidized iron-responsive element-binding proteins 2 (IRP2) ubiquitin ligase 1 (HOIL1), E3 enzyme HOIL1-interacting protein (HOIP) and adapter proteins SHANK-associated RH-domain interactor (SHARPIN). LUBAC modulates the complex I by mediating M1 linear ubiquitination of multiple components, including RIPK1, TRADD, NEMO, and TNFR1, which is important for the activation of NF-κB and mitogen-activated protein kinases (MAPKs) in cells stimulated by TNFα^9–11^. In addition, the activation of NF-κB promotes the expression of A20, a ubiquitin-modifying enzyme, which is also recruited into complex I to modulate the ubiquitination of RIPK1^12, 13^. A20 deficiency leads to increased K63 and decreased M1 ubiquitination in complex I^14^.

Parkin (Park2) is a RING-between-RING (RBR) E3 ubiquitin ligase. Loss-of-function mutations in Parkin are a major genetic cause associated with familial Parkinson’s disease (PD)^15^. The E3 ubiquitin ligase activity of Parkin has been shown to be involved in promoting the activation of NF-κB pathway by mediating degradation-independent ubiquitination of IKKγ/NEMO (NF-κB essential modifier) and TRAF2^16^. In addition, the expression of WT Parkin, but not pathogenic Parkin mutants, can transcriptionally up-regulate the expression of the mitochondrial guanosine triphosphatase OPA1 through NF-κB pathway to protect mitochondrial integrity and stress-induced cell death ^17^.

Although TNFα Signaling has been shown to be impaired in the absence of Parkin^17^, the interaction between Parkin and RIPK1 in mediating TNFα signaling has not been investigated before. Here we show that Parkin is recruited into complex I in response to TNFα signaling. Parkin interacts with RIPK1 and mediates K63 ubiquitination of RIPK1 on K376 site in complex I to activate NF-κB and MAPK signaling in cells stimulated by TNFα.

## Results

### Parkin interacts with the intermediate domain of RIPK1

**Fig. 1:**
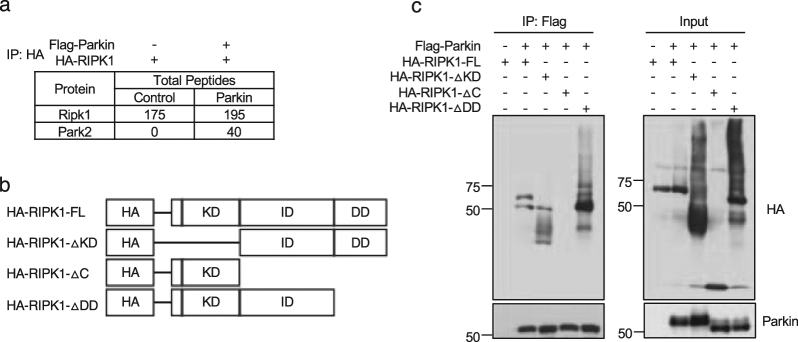
Parkin interacts with RIPK1. **a** 293 T cells were transfected with expression vectors of FLAG-tagged Parkin and HA-tagged RIPK1 for 24 h and then lysed with NP40 buffer and protease inhibitors with no EDTA. HA-tagged RIPK1 was immunoprecipitated with anti-HA antibody conjugated agarose and analyzed by mass spectrometry and quantified with LFQ module implemented in MaxQuant. The table listed the number of peptides from RIPK1 and Parkin identified in mass spectrometry analysis. **b** The expression vectors of HA-tagged full-length RIPK1, RIPK1- ΔKD, RIPK1- ΔC, and RIPK1- ΔDD were constructed. **c** The expression vectors of FLAG-tagged Parkin and HA-tagged full-length RIPK1, RIPK1-ΔKD, RIPK1-ΔC, and RIPK1-ΔDD were transfected into 293 T cells for 24 h. FLAG-Parkin was immunoprecipitated with anti-FLAG. The immunoprecipitates and cell lysates were analyzed by western blot with indicated antibodies

We first examined if Parkin and RIPK1 might interact. Because of the high number of cysteine residues (35) in Parkin and the requirement of zinc ions for its activity, our immunoprecipitation buffer contained fresh prepared reducing agent β-mercaptoethanol (0.1%) and no EDTA as recommended^18^. RIPK1 contains a kinase domain (KD) at the N-terminal part, an intermediate domain (ID) and a C-terminal death domain (DD)^1^. We expressed Flag-tagged RIPK1 and HA-tagged Parkin in 293 T cells and analyzed their interaction by mass spectrometry. From immunoprecipitated HA-RIPK1 from HEK 293 T cells expressing both RIPK1 and Parkin, mass spectrometry analysis detected significant number of peptides derived from Parkin (Fig. 1a), suggesting that RIPK1 can interact with Parkin. We next analyzed the domain of RIPK1 that binds with Parkin. We expressed FLAG-tagged Parkin, HA-tagged full length RIPK1 and three truncation mutants of RIPK1, HA-RIPK1-ΔKD, HA-RIPK1-ΔC, and HA-RIPK1-ΔDD in HEK 293 T cells to characterize their interactions by immunoprecipitation. We found that HA-RIPK1-FL, HA-RIPK1-ΔKD, and HA-RIPK1-ΔDD, but not HA-RIPK1-ΔC, could interact with Parkin (Fig. 1b, c). Thus, Parkin most likely interacts with the ID of RIPK1.

### Parkin mediates K63 ubiquitination of RIPK1

**Fig. 2:**
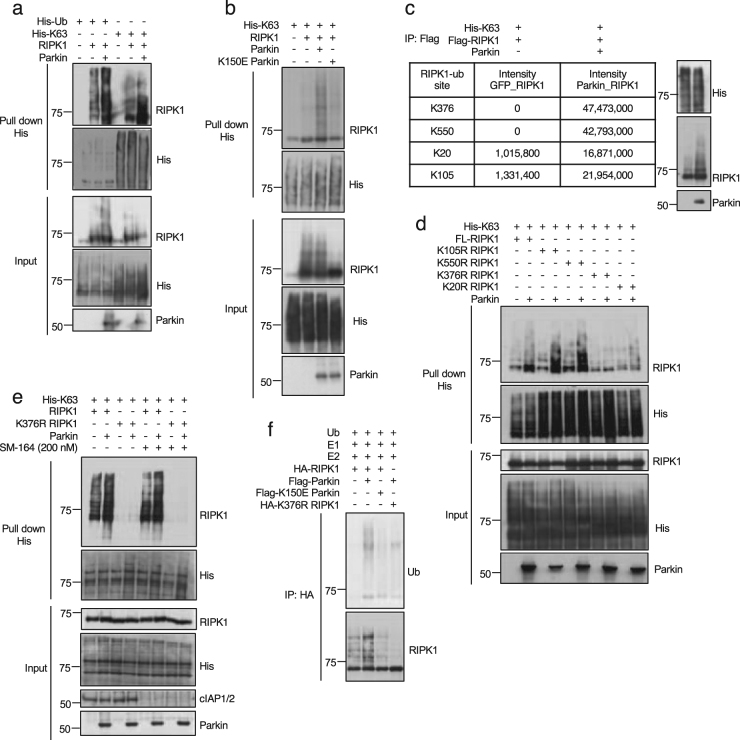
Parkin mediates K63 ubiquitination of RIPK1. **a** 293 T cells were transfected with expression vectors of RIPK1 and His-Ub or His-K63 ubiquitin with or without that of Parkin for 24 h and then lysed with 6 M urea. His-tagged proteins were pulled down with Ni-NTA. The pulled-down proteins and cell lysates were analyzed by western blotting with indicated antibodies. **b** 293 T cells were transfected with expression vectors of His-K63 ubiquitin, RIPK1 and Parkin or K150E Parkin for 24 h and then lysed with 6 M urea. His-tagged proteins were pulled down with Ni-NTA. The pulled-down proteins and cell lysates were analyzed by western blotting with indicated antibodies. **c** 293 T cells were transfected with expression vectors of His-K63 ubiquitin, Flag-RIPK1 with or without that of Parkin in 293 T cells for 24 h. Flag-tagged RIPK1 was isolated by immunoprecipitation with anti-FLAG agarose beads and analyzed for ubiquitination sites by mass spectrometry. Ubiquitinated Lys residues of RIPK1 identified and quantified by mass spectrometry are listed in the Table. **d** 293 T cells were transfected with expression vectors of His-K63 ubiquitin, K376R mRIPK1, K550R mRIPK1, K20R mRIPK1, K105R RIPK1 with or without that of Parkin as indicated for 24 h and then lysed with 6 M urea. His-tagged proteins were pulled down with Ni-NTA. The pulled-down proteins and cell lysates were analyzed by western blotting with indicated antibodies. **e** 293 T cells were transfected with expression vectors of His-K63 ubiquitin and WT RIPK1 or K376R RIPK1 with or without that of Parkin for 24 h and then treated with SM164 (200 nM) or DMSO for 4 h before lysed with 6 M urea. His-tagged proteins were pulled down with Ni-NTA. The pulled-down proteins and cell lysates were analyzed by western blotting with indicated antibodies. **f** 0.1 mM E1 UBE1, 1 mM E2 (UbcH7), 0.1 mM ubiquitin (Boston Biochem), 5 mM MgCl_2_, 2 mM ATP in 50 mM Tris-HCl (pH 7.5) mixed with Flag-Parkin or Flag-K150E Parkin immunoprecipitated with anti-FLAG, and HA-RIPK1 or HA-K376R RIPK1 immunoprecipitated with anti-HA from HEK 293 cells and incubated in a total volume of 50 μL for 30 min at 37 °C

Parkin can mediate K48 and K63 polyubiquitination^19^. To study if Parkin can mediate the polyubiquitination of RIPK1, we transfected expression vectors of His-Ub, His-K63 or His-K48 with that of RIPK1 and Parkin into 293 T cells and then pull down His-Ubi using Ni + beads. We found that WT Parkin could mediate K63 polyubiquitination of RIPK1 (Fig. 2a), but not K48 polyubiquitination of RIPK1 (Figure S1). K151E mutation in human Parkin impairs its ability to mediate ubiquitination^20^. We constructed homologous murine Parkin K150E mutant and found that K150E Parkin mutant lost the ability to mediate K63 polyubiquitination of RIPK1 (Fig. 2b).

We next determined the ubiquitination site of Parkin in RIPK1 by mass spectrometry. We immunoprecipitated Flag-tagged RIPK1 from HEK 293 T cells transfected with expression vectors of His-K63, Flag-RIPK1 and Parkin. Flag-tagged RIPK1 was isolated by immunoprecipitation and analyzed by mass spectrometry for ubiquitination. This analysis identified 4 lysine residues, K376, K550, K20 and K105, in mRIPK1 as possible ubiquitination sites by Parkin (Fig. 2c). We next analyzed and compared the ability of K376R, K550R, K20R, K105R RIPK1 mutants to be ubiquitinated by Parkin. We found that while co-expression of Parkin could still increase the ubiquitination of K550R, K20R, K105R mRIPK1, but not K376R mRIPK1 (Fig. 2d). Thus, Parkin can ubiquitinate K376 of mRIPK1.

Since K376 of mRIPK1 can be ubiquitinated by cIAP1^3^, we next examined if the ubiquitination of RIPK1 by Parkin could be affected upon depletion of cIAP1/2 by SM-164^21^. We found that the basal ubiquitination of RIPK1 was reduced with the treatment of SM-164; however, the ubiquitination of RIPK1 with co-expression of Parkin was minimally affected by SM-164 (Fig. 2e). On the other hand, K376R mutation eliminated both basal and Parkin-mediated RIPK1 ubiquitination. Thus, we conclude that while both cIAP1 and Parkin can ubiquitinate K376 mRIPK1 and furthermore, the ubiquitination of RIPK1 by Parkin can occur independent of cIAP1/2.

We further verified the ubiquitination of RIPK1 by Parkin in vitro. HA-RIPK1 and Flag-RIPK1 were isolated from 293 T cells transfected with their expression vectors individually and mixed with E1 and E2 in ubiquitination reactions. As shown in Fig. 2f, WT, but not K150E Parkin could promote ubiquitination of WT RIPK1. On the other hand, K376R RIPK1 could not be ubiquitinated by WT Parkin. From these results, we conclude that Parkin can directly ubiquitinate RIPK1 on K376.

### Parkin is recruited into complex I in RIPK1-dependent manner

**Fig. 3:**
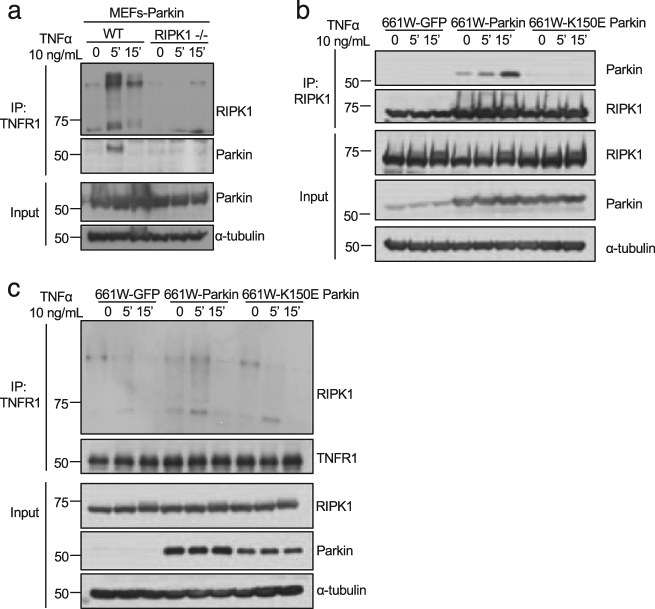
Parkin is recruited into TNF-RSC in RIPK1-dependent manner. **a** WT and Ripk1^−/−^ MEFs stably expressing Parkin were treated with TNFα 10 ng/mL for 0, 5, and 15 min. The cell lysates were immunoprecipitated with anti-TNFR1. The immunoprecipitates and cell lysates were analyzed by western blotting with indicated antibodies. **b** 661 W cells stably expressing GFP, Parkin or K150E Parkin were treated with TNFα10 ng/mL for 0, 5, and 15 min. The cell lysates were immunoprecipitated with anti-RIPK1. The immunoprecipitates and cell lysates were analyzed by western blotting with indicated antibodies. **c** 661 W cells stably expressing GFP, WT Parkin or K150E mutant Parkin were treated with TNFα 10 ng/mL for 0, 5, and 15 min. Complex I was immunoprecipitated with anti-TNFR1. The immunoprecipitates and cell lysates were analyzed by western blotting with indicated antibodies

We established Parkin-expressing wild type (WT) MEFs and Ripk1^−/−^ MEFs. Complex I was immunoprecipitated from both types of MEFs treated with TNFα using anti-TNFR1. The presence of Parkin in complex I could be detected at 5 min, but not at 0 and 15 min, in WT MEFs. However, the recruitment of Parkin into complex I was blocked in Ripk1^−/−^ MEFs (Fig. 3a). Thus, the recruitment of Parkin into complex I requires RIPK1.

We further characterized the interaction of Parkin and RIPK1 using 661 W cells stably expressing either WT or K150E mutant Parkin. We found that WT Parkin, but not K150E Parkin, interacted with RIPK1 in response to TNFα stimulation (Fig. 3b). Furthermore, the levels of RIPK1 ubiquitination in complex I were higher in 661 W cells expressing WT Parkin than that expressing K150E mutant Parkin (Fig. 3c). Thus, Parkin is involved in mediating K63 ubiquitination of RIPK1 on K376 in response to TNFα stimulation.

### Parkin promotes the recruitment of complex I components

**Fig. 4:**
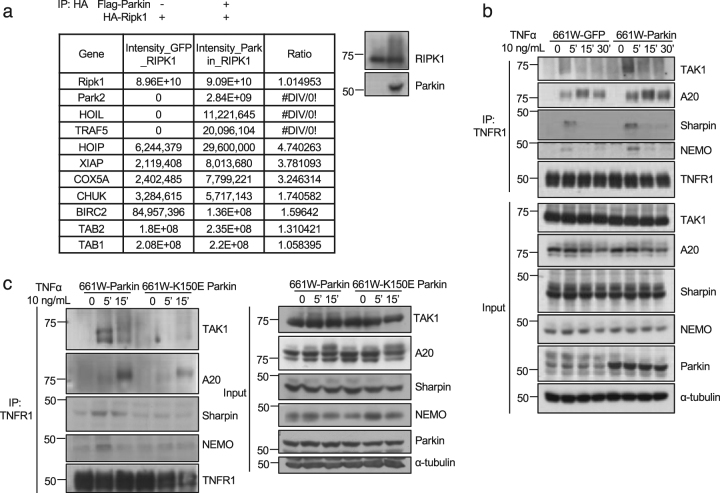
Interaction between Parkin and RIPK1 promotes the recruitment of components in complex I under TNFα stimulation. **a** The expression vectors of HA-tagged RIPK1 with or without FLAG-tagged Parkin were transfected into 293 T cells for 24 h. HA-RIPK1 was immunoprecipitated with anti-HA. The binding proteins of RIPK1 were identified by mass spectrometry and quantified with LFQ module implemented in MaxQuant. **b** 661 W cells stably expressing GFP or Parkin were treated with TNFα 10 ng/mL for 0, 5, 15 and 30 min. The cell lysates were immunoprecipitated with anti-TNFR1. The immunoprecipitates and cell lysates were analyzed by western blotting with indicated antibodies. **c** 661 W cells stably expressing Parkin or K150E Parkin were treated with TNFα 10 ng/mL for 0, 5 and 15 min. The cell lysates were immunoprecipitated with anti-TNFR1. The immunoprecipitates and cell lysates were analyzed by western blotting with indicated antibodies

The ubiquitination modification of RIPK1 is critical for the recruitment of multiple components in the complex I associated with TNFR1^22–25^. We thus investigated whether Parkin has any effect on the interaction of RIPK1 with other proteins. We transfected 293 T cells with expression vectors of RIPK1 with or without that of Parkin. Immunoprecipitated RIPK1 was analyzed by mass spectrometry to identify the interacting proteins of RIPK1. We found that with co-expression of Parkin, the levels of HOIL, HOIP, TAB1, TAB2 and cIAP2 (BIRC2) that interacted with RIPK1 were increased (Fig. 4a).

We next analyzed the recruitment of complex I in 661 W cells expressing vector alone or Parkin. We found that with Parkin expression, TNFα stimulated recruitment of TAK1, Sharpin, NEMO and A20 into complex I in 661 W cells were increased (Fig. 4b). On the other hand, the expression of Parkin K150E mutant was unable to promote the recruitments of these complex I components compared to that of WT Parkin (Fig. 4c). These results suggest that Parkin promotes the recruitment of complex I components in cells stimulated by TNFα.

### Parkin promotes the activation of NF-κB and MAPK signaling pathway

**Fig. 5:**
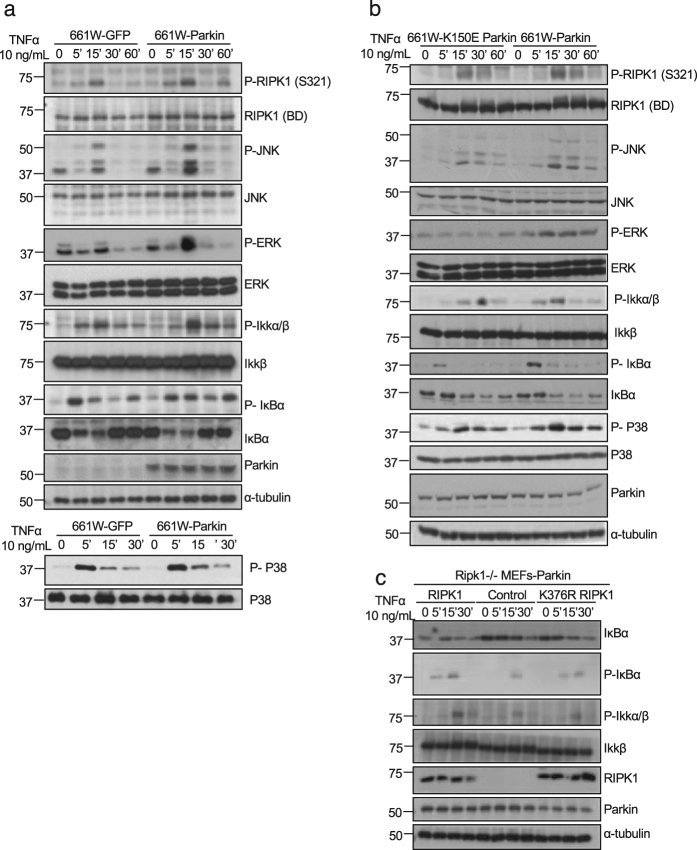
Interaction between Parkin and RIPK1 activate MAPK and NF-κB signaling under TNFα stimulation. **a**, **b** 661 W cells stably expressing GFP or Parkin (**a**) or Parkin or K150E Parkin (**b**) were treated with TNFα 10 ng/mL for 0, 5, 15, 30, and 60 min and then lysed with 6 M urea for western blotting with indicated antibodies. **c** Ripk1^−/−^ MEFs cells stably expressing Parkin were transfected with expression vectors of RIPK1 or K376 RIPK1, and treated with TNFα 10 ng/mL for 0, 5, 15, and 30 min. The cells were then lysed with 6 M urea for western blotting with indicated antibodies

Polyubiquitination of RIPK1 is important for the activation of NF-κB and MAPKs in TNFα stimulated cells^26, 27^. To investigate the effect of Parkin expression on the activation of NF-κB and MAPKs, we next analyzed the phosphorylation levels of Ser321 (S321) RIPK1 and IKKα/β in TNFα-stimulated 661 W cells expressing Parkin or GFP as a control. The results showed that the phosphorylation levels of both S321 RIPK1 and IKKs were increased in 661 W cells expressing Parkin compared to that of GFP (Fig. 5a). In addition, compared to that of WT Parkin, the expression of catalytically-inactive Parkin K150E mutant could not increase the levels of these phosphorylated proteins (Fig. 5b). Since the phosphorylation of S321 RIPK1 and IKKs are all mediated by TAK1^7, 8^, these results suggest that the expression of Parkin in 661 W cells increased the activation of TAK1 upon stimulation by TNFα.

Consistent with the increased activation of TAK1 and IKKs, the phosphorylation levels of IκBα were also increased. Furthermore, the levels of IκBα in Parkin-expressing cells were decreased (Fig. 5a). In addition, the activation of p38, JNK and ERK was also increased as indicated by their phosphorylation biomarkers in 661 W cells expressing Parkin.

To determine the importance of K376 RIPK1 in Parkin-mediated effect on NF-κB pathway, we complemented Parkin-expressing Ripk1^−/−^ MEFs with WT or K376R RIPK1. We found that the phosphorylation levels of Ikkα/β and IκBα induced by TNFα were lower in K376R RIPK1 expressing cells than that of complemented with WT RIPK1 (Fig. 5c). These results suggest that K376 RIPK1 is important for mediating the activation of NF-κB signaling by Parkin.

### Activation of NF-κB signaling by Parkin depends on RIPK1

**Fig. 6:**
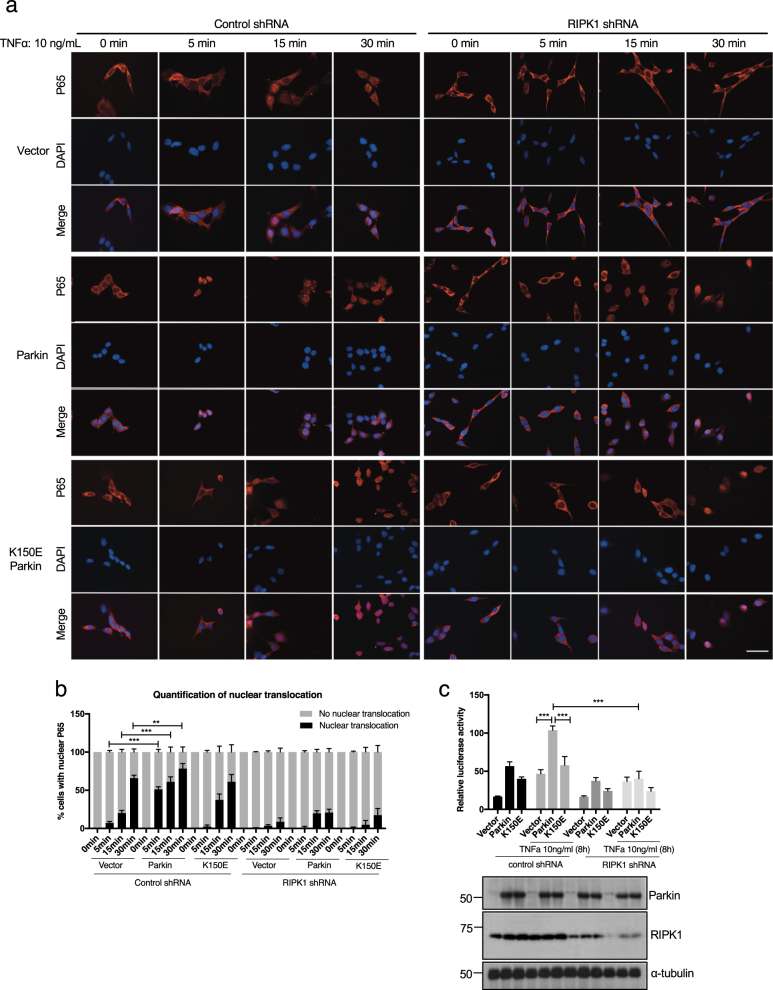
Interaction between Parkin and RIPK1 promotes NF-κB p65 nuclear translocation. **a** Nuclear translocation of NF-κB p65 was analyzed by indirect immunostaining in 661 W cell stably expressing vector, Parkin or K150E Parkin. These cells were transfected with control shRNA or RIPK1 shRNA for 24 h as indicated then treated with TNFα (10 ng/ml) for 0, 5, 15, and 30 min. Scale bars, 50 μm. **b** Colocalizations of p65 and DAPI were quantified in three independent experiments performed in duplicate. Data were represented as mean ± SEM. **c** 661 W cells stably expressing vector, Parkin or K150E Parkin were transfected with NF-κB luciferase reporter vector, CMV-Renilla (sea pansy) luciferase vector and control shRNA or RIPK1 shRNA as indicated for 16 h and then treated with or without TNFα (10 ng/ml) for 8 h as indicated. Luciferase activity in cell lysates was determined 24 h after transfection. The knockdown efficiency and expression levels were determined by western blotting. Data were represented as mean ± SEM of triplicates

The data above suggest that K63 ubiquitination of RIPK1 by Parkin can promote the activation of NF-κB in response to TNFα stimulation. We next investigated the effect of Parkin expression on the nuclear translocation of p65, a critical event in mediating the transcriptional response of NF-κB pathway by quantitatively measuring the nuclear translocation of p65 with TNFα stimulation in 661 W cells expressing WT or K150E mutant Parkin. We found that the expression of Parkin, but not K150E mutant, promoted the nuclear translocation of p65 in 661 W cells (Fig. 6a, b). On the other hand, the ability of Parkin to promote the nuclear translocation of p65 was compromised with RIPK1 knockdown (Fig. 6a, b).

**Fig. 7:**
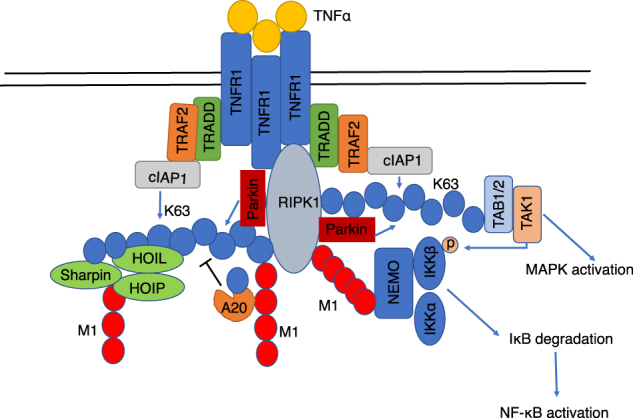
In response to TNFα, Parkin is recruited into complex I by interacting with RIPK1 to mediate its K63 ubiquitination on K376, which in turn promotes the recruitment of other complex I components, including TAK1, Sharpin, NEMO and A20, etc., to mediate the activation of NF-κB and MAPK.

In addition, we used the NF-κB promoter driven luciferase assay to determine the effect of Parkin on NF-κB activity induced by TNFα. We found that the expression of WT, but not K150E mutant, Parkin led to increased NF-κB driven luciferase activity induced by TNFα. Furthermore, knockdown of RIPK1 reduced both basal, as well as Parkin stimulated NF-κB activity induced by TNFα (Fig. 6c). Taken together, we conclude that the expression of Parkin promotes NF-κB activation induced by TNFα in RIPK1-dependent manner (Fig. 7).

Finally, we performed the cell viability assay to examine the effect of Parkin on cell death. The results showed that WT Parkin, but not K150E Parkin, demonstrated some protective effect on RIPK1-dependent apoptosis induced by TNFα with 5Z7 or SM-164 (Fig. S2a; Fig. S2b), but had no obvious effects on canonical apoptosis induced by TNFα with CHX (Fig. S2c). The expression of Parkin delayed necroptosis induced by TNFα, 5Z7 and Z-VAD in 661 W cells (Fig. S2d). The lack of strong protective effect by Parkin expression in these cellular models is likely because the effect of Parkin is primarily on the NF-κB pathway, and thus, the inactivation of NF-κB activation by protein synthesis inhibitor CHX, SM-164 and TAK1 inhibitor 5z7 reduces the protective effect of Parkin expression. The effect of Parkin on apoptosis and necroptosis needs to be investigated in future.

## Discussion

The scaffold function of RIPK1 has been shown by multiple studies to be involved in mediating the activation of NF-κB^3–5^. A subsequent study suggested that TNFα-induced NF-κB activation could occur in Ripk1^−/−^ MEFs^28^. This discrepancy, however, might be due to high concentration of TNFα (100 ng/ml) used in Wong et al.^28^ which might induce signaling through other receptors, e.g. TNFR2. Here we confirm the requirement for the scaffold function of RIPK1 in mediating TNFα-induced NF-κB signaling. Furthermore, we show that RIPK1 is involved in mediating the activation of NF-κB by Parkin. Previous studies have shown that Parkin activates NF-κB by promoting the ubiquitination of IKKγ/NEMO (NF-κB essential modifier) and TRAF2^16, 17^. Here we extend these studies to show that the interaction and ubiquitination of RIPK1 by Parkin is also involved in mediating the activation of NF-κB through the TNFR1-signaling pathway. Finally, K376 RIPK1 has been shown to be a ubiquitination site by cIAP1/2 [3]. Here we show that Parkin can also mediate K63 ubiquitination of RIPK1 on K376 independent of cIAP1/2. Thus, K376 RIPK1 may be ubiquitinated by multiple E3 ubiquitin ligases to regulate the activation of NF-κB. On the other hand, the expression of Parkin primarily delayed cell death in various cellular models of apoptosis of necroptosis. This is likely because Parkin primarily modulates the ubiquitination status of RIPK1 to control the activation of NF-κB, rather than directly inhibition of RIPK1 kinase activity as that of Nec-1s. The interaction of Parkin with RIPK1 in vivo should be investigated in future.

## Materials and methods

### Reagents and antibodies

The following reagents were used in this study: Recombinant mouse TNFα from Cell sciences (CRT192C) (Newburyport, MA, USA); and custom-synthesized Smac mimetic SM-164 by Selleckchem (Houston, TX, USA).

The following antibodies were used in this study: Parkin, Santa Cruz (sc-32282) (Dallas, TX, USA); FLAG-Tag, Cell Signaling Technology (2368) (Danvers, MA, USA); HA-Tag, Cell Signaling Technology (C29F4); RIPK1, Cell Signaling Technology (3493), and BD Biosciences (610459) (San Jose, CA, USA); His-probe, Santa Cruz (sc-8036); a-Tubulin, Sigma-Aldrich (T9026) (St Louis, MO, USA); TAK1, Cell Signaling Technology (5206); NEMO, Cell Signaling Technology (2685); p-IKKα/β, Cell Signaling Technology (2697); IKKβ, Cell Signaling Technology (8943); IκBα, Santa Cruz (sc-371); p-p38, Cell Signaling Technology (9211); p38, Cell Signaling Technology (9212); TNFR1, Cell Signaling Technology (13377S); A20, CST (5630); SHARPIN, Proteintech (14626-1-AP) (Chicago, IL, USA); NF-κB p65, Santa Cruz (sc-8008); RIPK1 polyclonal anti-p-S321 antibodies were produced in rabbits by Proteintech. The following secondary antibodies were used: anti-rabbit, SouthernBiotech (4050-05) (Birmingham, AL, USA), anti-mouse, SouthernBiotech (1031-05).

### Cell culture

661 W cells^29^, Human Embryonic Kidney Cells 293 T (HEK293T), and MEFs were cultured in Dulbecco’s modified Eagle medium (DMEM) (Gibco) supplemented with 10% (v/v) fetal bovine serum (FBS) (Gibco, Grand Island, NY, USA), 100 U/mL penicillin/streptomycin, and 1 mM sodium pyruvate (Invitrogen, Carlsbad, CA, USA). All cells were cultured under 5% CO_2_ at 37 °C and were tested for mycoplasma contamination by MycoAlert Mycoplasma Detection Kit (Lonza, Basel, Switzerland) every 3 months.

### Plasmids

Flag-tagged CDS domain of mouse Park2 cDNA was cloned into Lentiviral Vectors. HA-tagged CDS domain and truncations of mouse Ripk1 cDNAs were cloned into pMSCV vector. The single-site mutants were constructed by Mut Express® II Fast Mutagenesis Kit (Vazyme, Nanjing, China). The sequences were confirmed by DNA sequencing.

### Immunoprecipitation

The culture dishes or plates were cooled on ice and cells were washed three times gently with ice-cold PBS and lysed with NP40 lysis buffer for 20 min (min) at 4 °C. NP40 lysis buffer includes HEPES 25 mM, NP-40 1%, NaCl 400 mM, sucrose 0.3 M, fresh prepared β-mercaptoethanol 0.1%, Na3vo3 1 mM, NaF 50 mM, Protease Inhibitor mix (EDTA free) (1:100 from 100* stock) (Thermo, 88266, Waltham, MA, USA), NEM 50 mM, and PMSF (1:100). The cell lysates were centrifuged at 15,000 g for 20 min at 4 °C. Supernatants were incubated with antibodies overnight following by incubation with Protein A/G Agarose (Thermo) for 6 h at 4 °C. The agarose beads were washed four times with ice-cold NP40 lysis buffer. The immunoprecipitates were collected by boiling the agarose beads with SDS loading buffer for 5 min. The SDS loading buffer includes 2% SDS, 50 mM Tris-HCl (pH 6.8), 100 mM DTT and 10% glycerol.

### Lentivirus production and infection

Lentiviral Vector plasmids carrying Parkin cDNA were transfected into 293 T cells with packaging vectors including REV, VSVG and MDL using PEI for 6 h. After incubation for 48 h, the supernatant media of transfected 293 T were filtered using 0.45 µM membrane to collect lentivirus particles for infection.

### In-vitro ubiquitination assay

In vitro ubiquitination reaction reagents [0.1 mM E1 UBE1 (Boston Biochem, Boston, MA, USA), 1 mM E2 (UbcH7) (Boston Biochem) and 0.1 mM ubiquitin (Boston Biochem) in 5 mM MgCl_2_, 2 mM ATP, 50 mM Tris-HCl (pH 7.5)] were incubated with HA-tagged WT or K376R RIPK1 and Flag-tagged WT or K150E Parkin immunoprecipitated from HEK293T cell lysates in a total volume of 50 μL for 30 min at 37 °C.

### Immunocytochemistry

661W cells were plated on chamber slide (Lab-Tek, Rochester, NY, USA) and treated as indicated. Cells were fixed with 4% paraformaldehyde for 30 min and washed by PBS for three times, followed by blocking with 5% bovine serum albumin (Sigma), 5% goat serum (Sigma), 0.3% Triton X-100in and 0.1% Glycine (Thermo) in PBS for 30 min. Cells were then incubated with anti-p65 antibody (Santa Cruz, sc-8008) overnight at 4 °C and then were washed for 3 times by PBS following by incubation with fluorescent secondary antibody (Thermo, A-11029) for 1 h. After the incubation, cells were washed with PBS for three times and the nuclei were stained by DAPI (300 nM) (Thermo) following by the mounted with mounting media (Invitrogen, P36930). The images were acquired with a Yokogawa spinning disk confocal microscope (Nikon Ti fluorescence microscope).

### Dual Luciferase reporter assays

Plasmids encoding NF-κB promoter driven firefly luciferase and CMV driven Renilla luciferase were transfected into 661 W cells for 16 h and then treated with TNFα (10 ng/mL) for 8 h. The luciferase activity was determined luminometrically by using the dual luciferase assay system (Promega, Madison, WI, USA).

### Mass spectrometry and data analysis

The binding partners and ubiquitination sites of RIPK1 were identified by mass spectrometry. HA-RIPK1 or Flag-RIPK1 proteins isolated by immunoprecipitation from cell lysates were trypsin-digested on beads. The efficient enrichment of ubiquitinated peptides with diglycine remnant from digested peptide pools was accomplished by immunoprecipitation using anti-K-ε-GG antibody (PTM bio, Hangzhou, China). The resulted peptides were analyzed on Q Exactive HF Mass Spectrometer (Thermo). The identification and quantification of ubiquitinated peptides and proteins were done by MaxQuant^30^. The site localization scores were determined for confidently identified K-GG peptides using the AScore algorithm implemented in MaxQuant^31^. The tandem mass spectra were searched against UniProt mouse protein database together with a set of commonly observed contaminants. The precursor and fragment mass tolerance was set as 20 ppm. The cysteine carbamidomethylation was set as a static modification, and the oxidation of methionine as well as the ubiquitination of lysine (diglycine remnant) was set as variable modifications. The FDR at peptide spectrum match level and protein level were controlled below 1%. The quantification of proteins was done by the module of label free quantification in MaxQuant. The unique peptides plus razor peptides were included for quantification.

### Statistics

Each individual experiment was repeated at least three times. Statistics and graphs were performed by using Prism 7 and Microsoft Excel 2011. Data are expressed as the mean ± standard error of the mean (S.E.M.). Differences were considered statistically significant when *p* < 0.05 (*), *p* < 0.01 (**) or *p* < 0.001 (***).

## Electronic supplementary material

Supplementary figure legends

Supplementary fig 1

Supplementary fig 2
