# Supplementary material for: Parkin regulates NF-κB by mediating site-specific ubiquitination of RIPK1
Source: Cell Death Dis. 2018 Jun 28;9(7):732. doi: 10.1038/s41419-018-0770-z (PMC6023924; doi:10.1038/s41419-018-0770-z)
Supplement: Supplementary file 1 — Supplementary figure legends [file 41419_2018_770_MOESM1_ESM.docx]

**Supplementary Figure 1 Parkin cannot mediate K48 ubiquitination of RIPK1.**

293T cells were transfected with expression vectors of His-K48 ubiquitin and RIPK1 with or without Parkin for 24 h and then lysed with 6M urea. His-tagged proteins were pulled down with Ni-NTA. The pulled-down proteins and cell lysates were analyzed by western blotting with indicated antibodies.

**Supplementary Figure 2 The effect of Parkin expression on cell death.** (a) 661W cells stably expressing GFP or Parkin or K150E Parkin were pretreated with Nec-1s (20μM) or DMSO for 1 hour and then treated with TNFα 10ng/mL + SM164 50nM for indicated periods of time. (b) 661W cells stably expressing GFP or Parkin or K150E Parkin were pretreated with Nec-1s (20μM) or DMSO for 1 hour and then treated with TNFα 10ng/mL + 5Z7 0.5μM for indicated periods of time. (c) 661W cells stably expressing GFP or Parkin were treated with TNFα 10ng/mL + CHX 1ug/mL for indicated periods of time. (d) 661W cells stably expressing GFP or Parkin or K150E Parkin were treated with TNFα 10ng/mL + 5Z7 0.5μM + Z-VAD 20μM for indicated periods of time. The cell viability was determined using CellTiterGlo.
