# Supplementary figures and images for: Parkin regulates NF-κB by mediating site-specific ubiquitination of RIPK1
Source: Cell Death Dis. 2018 Jun 28;9(7):732. doi: 10.1038/s41419-018-0770-z (PMC6023924; doi:10.1038/s41419-018-0770-z)

# Supplementary Figure 1

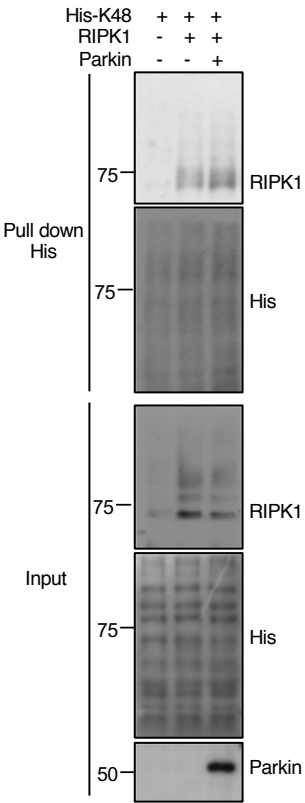

Supplement: Supplementary file 2 — Supplementary fig 1 [file 41419_2018_770_MOESM2_ESM.pdf]

# Supplementary Figure 2

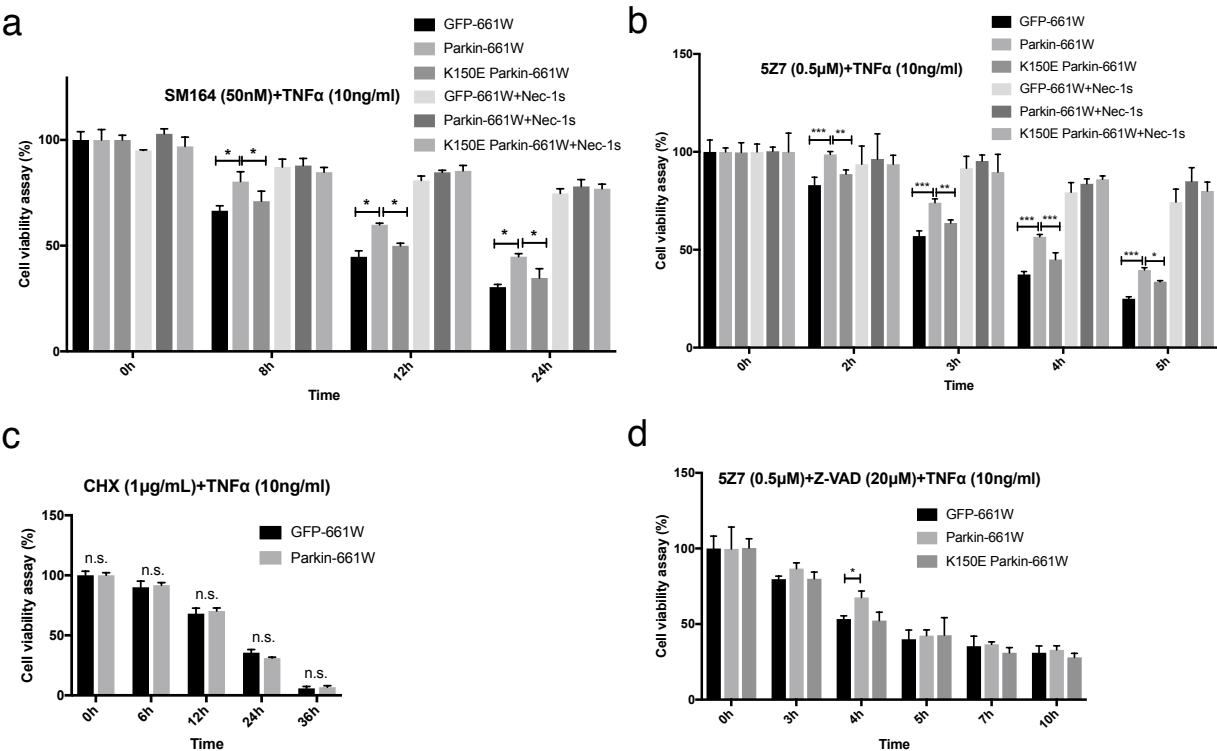

Supplement: Supplementary file 3 — Supplementary fig 2 [file 41419_2018_770_MOESM3_ESM.pdf]
